# XMetDB: an open access database for xenobiotic metabolism

**DOI:** 10.1186/s13321-016-0161-3

**Published:** 2016-09-15

**Authors:** Ola Spjuth, Patrik Rydberg, Egon L. Willighagen, Chris T. Evelo, Nina Jeliazkova

**Affiliations:** 1 Department of Pharmaceutical Biosciences and Science for Life Laboratory, Uppsala University, 75124 Uppsala, Sweden; 2 Department of Drug Design and Pharmacology, University of Copenhagen, Universitetsparken 2, 2100 Copenhagen, Denmark; 3 Department of Bioinformatics - BiGCaT, Maastricht University, P.O. Box 616, UNS50 Box 19, 6200 MD Maastricht, The Netherlands; 4 IdeaConsult Ltd, 4 A.Kanchev str., 1000 Sofia, Bulgaria

**Keywords:** Xenobiotic, Metabolism, Database, Cytochrome, P450

## Abstract

Xenobiotic metabolism is an active research topic but the limited amount of openly available high-quality biotransformation data constrains predictive modeling. Current database often default to commonly available information: which enzyme metabolizes a compound, but neither experimental conditions nor the atoms that undergo metabolization are captured. We present XMetDB, an open access database for drugs and other xenobiotics and their respective metabolites. The database contains chemical structures of xenobiotic biotransformations with substrate atoms annotated as reaction centra, the resulting product formed, and the catalyzing enzyme, type of experiment, and literature references. Associated with the database is a web interface for the submission and retrieval of experimental metabolite data for drugs and other xenobiotics in various formats, and a web API for programmatic access is also available. The database is open for data deposition, and a curation scheme is in place for quality control. An extensive guide on how to enter experimental data into is available from the XMetDB wiki. XMetDB formalizes how biotransformation data should be reported, and the openly available systematically labeled data is a big step forward towards better models for predictive metabolism.

**Electronic supplementary material:**

The online version of this article (doi:10.1186/s13321-016-0161-3) contains supplementary material, which is available to authorized users.

## Background

Xenobiotic metabolism covers the biochemical modification of drugs and xenobiotics by living organisms. These biotransformations are usually carried out by specialized enzymatic systems such as the cytochrome P450s and the UDP-glucuronosyltransferases [1], with the aim to make compounds more soluble and more easily excreted from the body.

Understanding how xenobiotic metabolism occurs in the human body is important in two fields particularly: drug discovery and toxicology. In drug discovery, one needs to understand what metabolites of a potential drug are formed to be able to study what effects they have [2]. Occasionally, some metabolites are more potent than the drug itself. For example, cyclophosphamide is a prodrug that is implicated to be activated by P450s [3]. Furthermore, metabolism can cause unwanted side effects, for example by interfering with the potential use of other drugs by inhibiting certain enzymes and, for example, change blood concentrations of drugs [4]. In the field of toxicology, understanding the metabolism of all types of chemicals is important, even if the compounds themselves are not toxic, and it is their metabolites that cause the toxic effects. This also makes it important to understand metabolism when building in silico models that predict toxicity, as the molecular properties of the original compound and its metabolites may differ significantly. The evaluation of the metabolic fate and metabolism similarity of target and analog compounds in the context of read-across is an essential part of the framework for toxicological assessment proposed by Wu et al. [5] and relevant methods and tools are emphasized in a recent review on in-silico approaches for predicting toxicity [6].

The creation and validation of in silico models for predicting the metabolism of xenobiotics is an active field of research [7–17]. However, the available of suitable data is limited in two ways. First, relevant data used to construct these models are either locked away in proprietary and commercial databases, such as the Accelrys Metabolite database (http://accelrys.com/products/collaborative-science/databases/bioactivity-databases/biovia-metabolite.html), or is put together specifically for each new model [14]. The availability of data in a public database accessible in an open format and curated by the scientific community would be a major step forward in decreasing the work required to create new models, and to enable comparisons of different models.

Second, the experiments to accurately link the metabolic conversion to a specific enzyme are rare. Often microsomes and hepatocytes of animal and human origin are used [18], in which it cannot always fully be certain which enzyme really does the conversion. Currently, the most detailed experimental procedures used to verify which metabolites are formed use cDNA-expressed drug-metabolizing enzymes, followed by LC/MS analysis of the formed metabolites [19]. A further complication is that even when reference compounds of the metabolites are available in such studies, it is still not always possible to accurately identify at which atomic position the conversion happens, for example, of an aromatic ring hydroxylation reaction. Other types of experiments give information about the metabolism, but few studies give the full picture. Nevertheless, it is this precise recording of experimental detail is critical, whatever the used method is.

Published data on xenobiotic metabolism, which may serve as an information source of a new database, are currently fragmented over many journals and publications, using different experiment types, and provided in different formats, conforming to different standards. There are several existing databases that contain information relevant for xenobiotic metabolism, including DrugBank [20], SuperCYP [21], hDBMdb [22], Metrabase [23], Human Metabolome Database (HMDB) [24] and Transformer [25]; Drugbank reports substrate—product—enzyme, and while it includes references, these are typically to other database rather than primary literature; Transformer reports substrate—enzyme—reference, but not what the product is; Metrabase focuses on transporters and xenobiotic metabolism; HMDB lists the enzyme and literature but not the species the transformation was observed in, and, SuperCYP reports substrate—product—enzyme. Importantly, these databases do not include atom–atom mapping which is crucial for building predictive models for site-of-metabolism. hDBMdb does not seem to be operational anymore. Further, none of the previously mentioned databases has an open application programming interface (API) for direct consumption in scripts and third party applications, making them less useful for large scale modeling [26–29]

Further, the commercially available databases are very expensive and sometimes contain unpublished data, leading to further literature studies to find validation data that can be used in publications. Also, since different formats and standards are used across different labs, there is no consistent evaluation system for these kinds of models, resulting in published statistics that are not directly comparable between publications, e.g. mapping from mechanism to atoms that have not been performed in an identical fashion.

This lack of publicly available data has led to numerous repetitive literature searches for many academic research groups interested in metabolite prediction modeling. However, finding appropriate literature with enough detail is a challenge in itself. The knowledge about biotransformations is limited and reports about it are scattered. Peer-reviewed literature is not the only source of information, nor does literature report all the details we want to capture. Anecdotal examples even shows literature citing conference posters and package inserts as primary sources [30]. Other literature includes FDA submissions, but these too can provide only limited detail and may also lack information about products formed.

We here present XMetDB—an open access database for xenobiotic metabolism implemented as an online system for deposition and sharing of experimental data. It is the first database that contain atom reaction center mapping and also includes a new reporting standard for this, with data and software available under Open licenses.

## Results

XMetDB consists of a database, an application layer for interacting with the database, and a user interface. The core data stored in the database are Observations, which consists of an experimentally detected enzyme-catalyzed reaction of a substrate that yields a product. XMetDB contains the chemical structure of substrates and products, and also includes annotations of atoms which are affected by the metabolizing reaction, indicating experimentally derived site-of-metabolism annotations. The experimental conditions are limited to the type of experiment (one of enzymes, hepatocytes and microsomes) and enzymes involved. The species assumed is human. The initial design does not allow specifying the species, as the intention was to built a database for human metabolism. Associated structured information includes uncertainty of the atom mappings (either certain or uncertain), amount of product formed in the reaction (one of major, minor, or unknown), a literature reference, and comments as free text. Enzymes, such as members of the Cytochrome P450 family, can be added separately and are linked out to UniProt via UniProt ID.

In addition, each observation can be tagged as curated or not, and assigned free text comment.

All Observations have unique identifiers, for example the arbitrarily chosen entry XMETDB153 for the observation of the P450-mediated oxidation of quinoline into 3-hydroxyquinoline by the CYP2E1 isoform, and comprises a metabolic transformation (substrate, product), the enzyme, a literature reference, and a categorization of the experiment with the enzyme identified by its name and UniProt identifier. The reference in the example is provided as free text, while increasingly the DOI is given instead of a textual reference.

Version 1.0 of XMetDB contains 162 observations from 21 scientific papers from 14 journals, covering 117 chemical structures and 95 enzymes.

### Web interface

XMetDB provides two interfaces: an HTML-based interface for access via a web browser and aimed at humans, and an API set up for programmatic interaction. The key features of the interfaces are: (1) browse and search for observations, (2) submit new observations, and (3) add and browse enzymes. The API is described later, and we first explain the graphical user interface.

#### Data querying

**Fig. 1:**
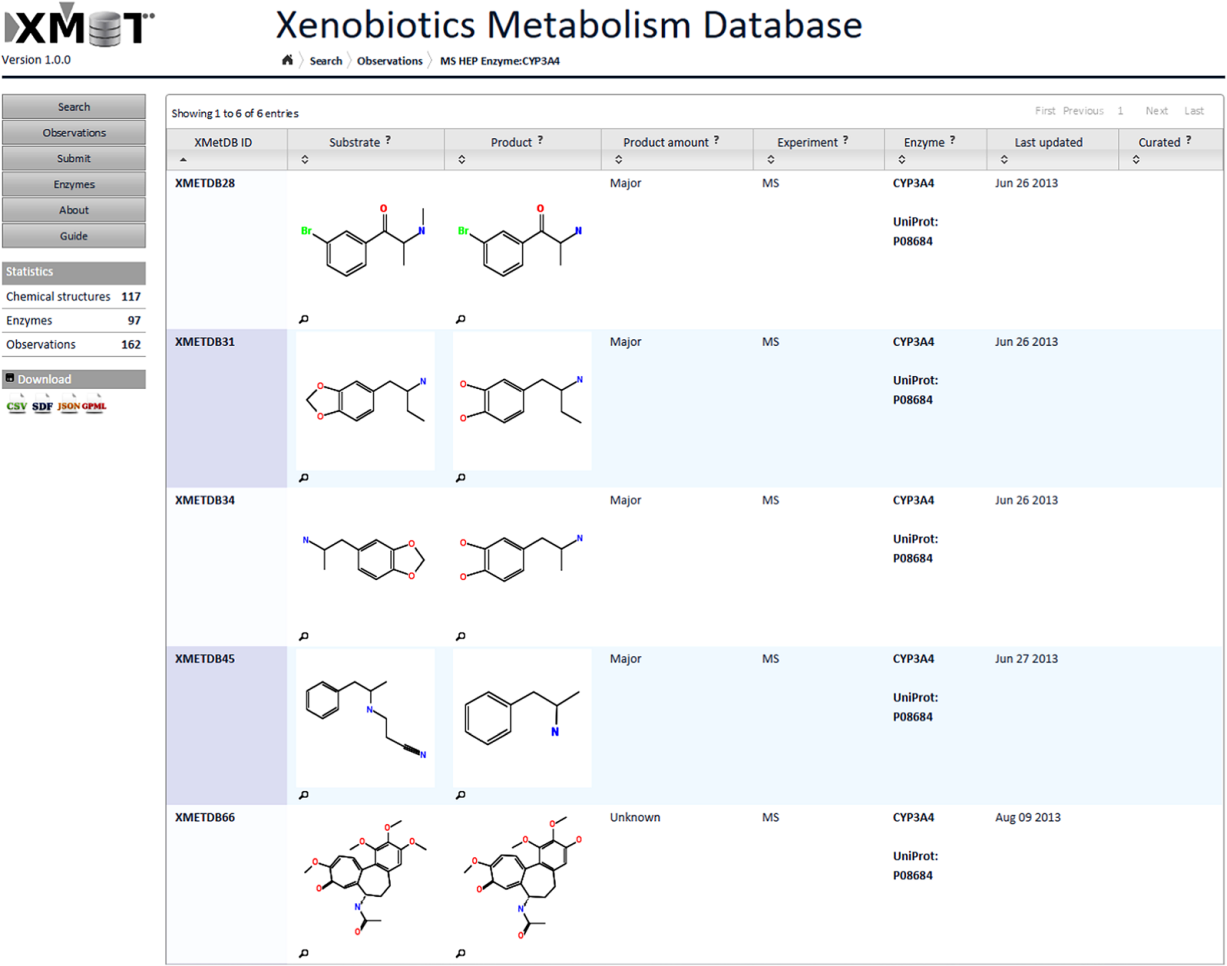
Browsing observations in XMetDB. When browsing all or a subset of observations, the table shows the chemical structures of substrate and product together with other data and metadata

**Fig. 2:**
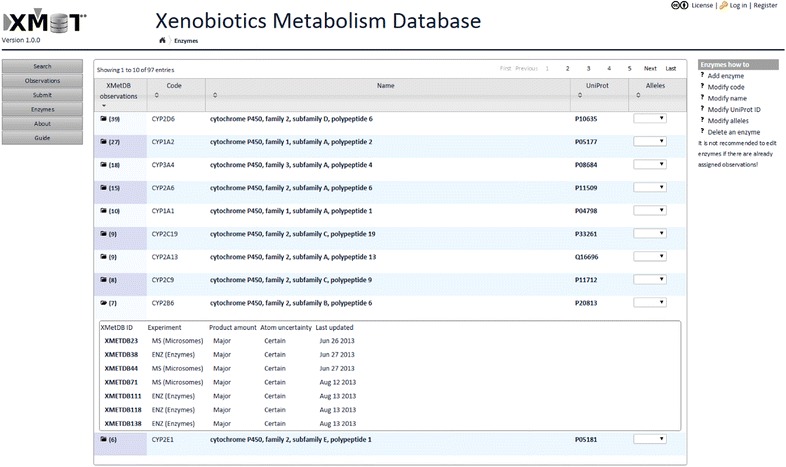
Browsing enzymes in XMetDB. When browsing all or a subset of enzymes, the table shows the Code, Name, UniProt ID, and optionally allele for each entry

The principal way to browse the data in XMetDB is by listing all observations (see Fig. 1 for an example). The list shows observations paginated, and the number of observations shown can be changed from 10 to 20, 50, 100, and all observations. Alternatively, it is possible to browse the observations by enzyme, presenting a list of observations for a particular enzyme (see Fig. 2 for an example). Both lists can be sorted by any of the available columns and lead to individual observations, each with a unique identifier.

The user interfaces in XMetDB supports searching for observations by status, experiment type, enzyme, reference, XMETID, and allele. It is also possible to search for substrates and products by chemical similarity and substructure using a structure diagram editor or by chemical identifier (CAS, Chemical Name, SMILES, InChI or SMARTS for a substructure search). The resulting list of structures is ordered by similarity, clicking the folder icon returns a list of observations involving this structure.

#### Data entry

**Fig. 3:**
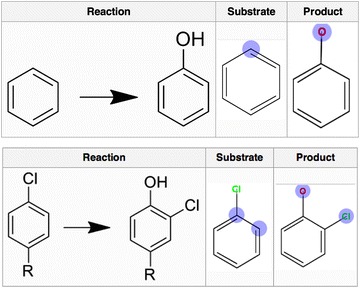
Examples of how biotransformations should be reported. This example shows the atoms defined as reaction centra indicated in *blue*. *Top* Hydroxylation of aromatic carbons. *Bottom* Hydroxylation of aromatic carbons coupled with halogen migration

Submission of a new observation works as a two-step procedure: (1) Enter substrate and product structures and meta-data describing the experiment; (2) Select atom mappings for the reaction, which is included to enable more accurate site-of-metabolism modeling. All atom mappings used in XMetDB should be annotated as either Certain or Uncertain, where certain means that as far as possible it is known at which atom (or atoms) the reaction is initiated. To help the users, a large collection of metabolic reactions and their corresponding sites of metabolism have been collected, and are available in the XMetDB wiki: http://www.xmetdb.org/wiki/, together with an extensive guide for how to enter experimental data. An example of hydroxylation of aromatic carbon atoms by cytochromes P450 is shown in Fig. 3.

#### Data curation

XMetDB supports a curator role, with the purpose to ensure high quality data in the database. Data that has been verified by a curator besides the original submitter has been validated by at least one additional person, and the curator has been verified by the administrators as an expert on biotransformations for at least one enzyme family. The curators can edit all observations but not essential info such as experiment and enzymes. The curators can change atom highlighting and comments and typos in references. The curators can set the flag curated to yes for any observations. The users may indicate their availability to act as a curator, but the role must be assigned by an administrator.

#### Data export

Lists of observations, such as the results of a query, can be exported as column separated values (CSV) or structure data file (SDF) formats (see Table 1 for details). CSV files include the observations along with chemical structures represented as SMILES and InChI, with column names as listed in Table 2. SDF files includes chemical structures with the observations data and metadata stored as a set of SDF tags (an example file is given in Additional file 1). Single reaction entries can be exported in the GPML format, which is the XML language used by WikiPathways for storage of pathways.

**Table 1:** Download formats

Format	Media type	HTTP header	URI parameter
CSV	text/csv	Accept: text/csv	?media=text/csv
SD file	chemical/x-mdl-sdfile	Accept: chemical/x-mdl-sdfile	?media=chemical/x-mdl-sdfile

**Table 2:** Export column headers

Name	Description
URI	XMetDB observation URI
Identifier	XMetDB observation identifier
Substrate SMILES	SMILES of the substrate structure
Substrate InChI	InChI of the substrate structure
Product SMILES	SMILES of the product structure
Product InChI	InChI of the product structure
Product amount	The product amount. One of Major, Minor or Unknown
Experiment type	The experiment. One of Hepatocytes, Microsomes, Enzymes
Enzyme code	Enzyme code
Enzyme name	Enzyme name
UniProt	Enzyme UniProt ID
Reference	The publication from which the data is taken, either as a DOI or as a plain text reference
Comment	Free text comment

#### Web API

The XMetDB database exposes an open Application Program Interface (API) that allows the data to be accessed programmatically by software applications (e.g. workflow engines, scripts) and other web services [31].

The main XMetDB API components are observations, enzymes, and chemical structures. Every component is assigned an unique URI, e.g. http://xmetdb.org/protocol/XMETDB1 for an observation. The API follows RESTful principles, where each component (the term in the REST vocabulary is a “resource”) allows only four operations:

- GET—to retrieve the content of the resource
  e.g. GEThttp://xmetdb.org/protocol/XMETDB1
- PUT—update the content of the resource
  e.g. PUT (some data to) http://xmetdb.org/protocol/XMETDB1
- POST—-create a new resource
  e.g. POST (representation of a new resource to) http://xmetdb.org/protocol, the result is e.g. http://xmetdb.org/protocol/XMETDB2
- DELETE—remove the resource
  e.g. after DELETEhttp://xmetdb.org/protocol/XMETDB1 this resource will no more exist

The Web API is described extensively in XMetDB Wiki at: http://www.xmetdb.org/wiki/API.

### Implementation

XMetDB is implemented as a Java Web application (source code at https://github.com/xmetdb/xmetdb-server), which is accessible via a web browser and programmatically via a REST API. The web application is using the Restlet framework (http://restlet.com/projects/restlet-framework/) and is deployable in a compatible servlet container. The XMetDB user interface is built with FreeMarker templates http://freemarker.incubator.apache.org/ and JavaScript, interacting with the server side and the database through the REST API. The experimental data is stored in a MySQL database, the database schema is available at https://github.com/xmetdb/xmetdb-server/blob/master/xmet-db/src/main/resources/org/xmetdb/xmet/protocol/db/sql/xmetdb.sql.

The core requirement to store and query chemical structures and allow interactive editing of atom–atom mapping is enabled through interaction with a separate web application (AMBIT 2.4.11 [32]), which provides a web service interface to a chemical structure database. The AMBIT package enables upload or retrieval of individual structures or entire datasets, searching by identifiers, similarity, and substructure [33]. In AMBIT, each XMetDB observation corresponds to a dataset, where the substrate and the products are stored. The atom–atom mapping are stored as OpenTox features, unique to the ”observation” dataset and associated with the chemical structure [32]. AMBIT follows the REST approach to representation of all resources, for example the chemical structure diagram can be retrieved by requesting the proper MIME type (i.e. image/png) of a chemical structure resource. The structure diagram rendering was extended to generate an image map along with the image, delineating the atoms. The image map is used to display highlighted atoms and enables the interactive atom selection. An alternative implementation with SVG rendering was considered, but was not selected, as at the beginning of XMetDB implementation the browser SVG support was limited. As a structure diagram editor the freely available ChemDoodle 2D Sketcher [34] is used (JavaScript). The 2D Sketcher is embedded in a separate frame, as a workaround of the conflicting version of jQuery dependencies at the time of the development.

From a deployment perspective, XMetDB consists of two web containers: xmetdb.war and ambit2.war, available both as pre-deployed online web services at http://xmetdb.org, and as a downloadable web application which can be deployed in a compatible servlet container. The chemical structures and datasets are represented as described in [33], enhanced with JSON representation to facilitate the user interface implementation (http://ambit.sourceforge.net/api.html). The supported data formats are JSON and RDF (RDF/XML and RDF/N3) and asynchronous jobs are handled according to OpenTox API version 1.2 specifications [32].

#### Users and roles

The XMetDB application supports the following user roles: (1) regular user, (2) curator and (3) administrator. A regular user is any registered user who is logged in; and can add observations to the database, save searches as alerts, edit the user profile, and flag availability as curators. A curator is a regular user who has been approved as a curator. Beyond regular user rights, a curator can approve that data submitted by other users has been added in a consistent manner, and verify that the atom mappings and references are correct. As part of the curation process he/she can also edit atom mappings, and metadata of an observation. An administrator is a curator with additional rights (to grant the curator role to other users, to modify the enzyme list, delete observations, modify user information etc.). Searching and browsing the observations as well as exporting data through the web interface or API does not require the user to be logged in.

## Discussion and conclusions

Extracting biotransformation data from literature is a cumbersome process, and crowdsourcing initiatives are needed in order to propel scientific discoveries and enable computational model building. The aim of XMetDB is to provide scientists with a public repository where xenobiotic metabolism data can be uploaded, shared, and integrated with other observations. Other databases related to metabolism do not include atom–atom mapping, and in this manuscript we propose a formalization of how xenobiotic metabolism data should be reported in order to improve computational model building. We acknowledge here, however, that current experimental methods may not always provide all the detail we ask to be reported. The open access philosophy of XMetDB allows for any content to be uploaded, and a curation system allows for curators to ensure that the database contents are of high quality. XMetDB hence provides the means for the community to collectively build up a knowledge base over time, and it is our hope that the community will adopt the system and deposit annotated metabolism data upon publication in scientific journals. For the future we also envision to import reaction data from other databases or possibly via text mining, which could encourage reporting and investigations on atom–atom mapping.

The advanced search functions of XMetDB allow for querying substrates and products by chemical structure and various types of metadata using the web portal and also the programmatic API from third party applications and scripts. An example of an external collaborator that has chosen to support XMetDB is the WikiPathways project [35], which contributed code to support exporting data in GPML format [36]. The implementation with an available REST API with JSON serialization enables building graphical summaries of the data, development of JavaScript widgets, programmatic interaction and user interfaces beyond the current XMetDB web pages implementation.

The publicly available systematically labeled data in XMetDB will be a big step forward towards improved models for predictive metabolism. Future plans include to integrate XMetDB in Bioclipse [37] and expand the content with more data.

## Additional file

10.1186/s13321-016-0161-3 Example of exported SDF file from XMetDB.
